# Prenatal Care and Perinatal Regionalization for Congenital Heart Defects

**DOI:** 10.1001/jamanetworkopen.2025.42135

**Published:** 2025-11-09

**Authors:** Christina Laternser, William A. Grobman, Cecilia Albaro, Brett R. Anderson, Beau Batton, Lynn M. Yee, Joyce L. Woo

**Affiliations:** 1 Division of Pediatric Cardiology, Department of Pediatrics, Ann & Robert H. Lurie Children’s Hospital of Chicago, Chicago, Illinois; 2 Division of Maternal-Fetal Medicine, Department of Obstetrics and Gynecology, The Warren Alpert Medical School of Brown University, Providence, Rhode Island; 3 Division of Pediatric Cardiology, Department of Pediatrics, University of Illinois College of Medicine, Peoria; 4 Division of Pediatric Cardiology, Department of Pediatrics, Icahn School of Medicine at Mount Sinai, New York, New York; 5 Department of Population Health Science & Policy, Icahn School of Medicine at Mount Sinai, New York, New York; 6 Center for Child Health Services Research, Mindich Child Health and Development Institute, Icahn School of Medicine at Mount Sinai, New York, New York; 7 Division of Neonatology, Department of Pediatrics, Southern Illinois University School of Medicine, Springfield; 8 Division of Maternal-Fetal Medicine, Department of Obstetrics and Gynecology, Northwestern University Feinberg School of Medicine, Chicago, Illinois; 9 Department of Medical Social Sciences, Northwestern University Feinberg School of Medicine, Chicago, Illinois

## Abstract

**Importance:**

Regionalization is a system by which neonates with congenital heart defects (CHDs) are directed to risk-appropriate levels of care. However, the role of prenatal care in delivery location remains unclear.

**Objective:**

To estimate associations between prenatal care adequacy and the likelihood of delivery at a pediatric cardiac center.

**Design, Setting, and Participants:**

This cross-sectional study examined retrospective data from the Illinois Department of Public Health’s Adverse Pregnancy Outcomes Reporting System. Participants included neonates born with CHDs in Illinois from 2013 to 2021. Data were analyzed from November 2024 to May 2025.

**Exposures:**

Two binary exposure variables: (1) prenatal care initiation—none vs inadequate prenatal care (initiated after the fourth month or less than 50% of recommended visits) and (2) prenatal visit frequency—intermediate (50% to 79% of recommended visits) vs adequate (80% to 109% of recommended visits) or adequate plus (110% or more of recommended visits) prenatal care.

**Main Outcomes and Measures:**

Delivery at a pediatric cardiac center was the main outcome. Multivariable linear probability models estimated associations between prenatal care and delivery hospital, controlling for demographic and clinical characteristics. Regressions were stratified by CHD severity (eg, mild, moderate, and severe), as severe defects require intervention at a cardiac center within the first week of life.

**Results:**

Of 12 113 neonates with CHD, 3076 (25.4%) were born at a cardiac center and 1579 (13.0%) had severe CHD. Distribution for prenatal care initiation was: 272 (2.3%) had no prenatal care and 1617 (13.4%) had inadequate prenatal care. Distribution for prenatal visit frequency was: 1304 (10.8%) had intermediate prenatal care, 4217 (34.8%) had adequate prenatal care, and 4703 (38.8%) had adequate plus prenatal care. Prenatal care initiation was associated with a 10.5 (95% CI, 4.7 to 16.2) percentage point higher probability of delivery at a cardiac center for those with fetuses who had mild CHD and 30.2 (95% CI, 13.6 to 46.9) percentage point higher probability for severe CHD. For mild CHD, adequate plus prenatal care was associated with a lower probability of delivery at a cardiac center by 6.7 (95% CI, −4.0 to −9.4) percentage points compared with intermediate prenatal care. Prenatal visit frequency was not associated with delivery at a cardiac center for severe CHD.

**Conclusions and Relevance:**

In this cross-sectional study, delayed prenatal care initiation was associated with higher probability of delivery at a cardiac center, especially for severe CHD. More prenatal visits may help appropriately direct mild cases to noncardiac centers. Operationalization of regionalized CHD care requires consideration of many factors, including prenatal events.

## Key Points

**Question:**

What is the association between prenatal care initiation, prenatal visit frequency, and delivery location for neonates with congenital heart defects (CHDs)?

**Findings:**

In this cross-sectional study of 12 113 neonates born with CHDs in Illinois, delayed prenatal care initiation was associated with increased probability of delivery at a cardiac center compared with no prenatal care. Furthermore, for those with mild CHDs, more prenatal visits were associated with a 6.7 percentage point decrease in probability of delivery at a cardiac center.

**Meaning:**

These findings suggest that prenatal care initiation and visit frequency may both influence risk-appropriate care among neonates with CHDs.

## Teaser

This cross-sectional study examines the associations between prenatal care adequacy and the likelihood of delivery at a pediatric cardiac center in Illinois.

## Introduction

Congenital heart defects (CHDs) are the most common and resource-intensive birth defects in the US.^1^ Among those who require cardiac surgery, approximately 15% to 20% have severe CHDs such as hypoplastic left heart syndrome,^2,3^ which account for more than 3 trillion US dollars in direct and indirect costs annually^4^ due to highly specialized care needs, beginning with specific delivery plans^5^ and high-risk neonatal intervention at a cardiac center.^6^ In contrast, fetuses with mild CHD, such as septal defects, can be safely delivered at centers without capabilities for cardiac intervention, which can still include high-level neonatal intensive care for noncardiac comorbidities such as prematurity.^7^ This difference in clinical need underscores the importance of regionalization of perinatal care, ie, directing neonates with CHDs to hospitals equipped for their specific needs.^8^

Prenatal care is key to regionalization as it facilitates early diagnosis—up to 70% of severe CHDs are diagnosed prenatally in some US regions^9,10^—allowing for delivery at a pediatric cardiac center for these individuals.^11,12^ Yet, crucial knowledge gaps remain: can delayed prenatal care increase the likelihood of delivery at a cardiac center for those with severe CHD, compared with no prenatal care? Do more prenatal visits influence this likelihood?

To address these questions, we used a statewide administrative database to estimate how 2 components of prenatal care adequacy—initiation of care and frequency of visits—are associated with delivery location for neonates with CHDs. We focus on delivery location rather than clinical outcomes (eg, length of stay, morbidity, mortality) since these reflect surgical effects rather than effects of regionalization itself. We hypothesized that initiating prenatal care, even if delayed, increases the probability of delivery at a cardiac center for neonates with severe CHD, and that more prenatal visits further enhance this probability.

## Methods

### Ethical Oversight

The institutional review boards of Ann & Robert H. Lurie Children’s Hospital of Chicago and the Illinois Department of Public Health approved this study. This analysis follows the Strengthening the Reporting of Observational Studies in Epidemiology (STROBE) reporting guideline. Consent was not required because there was no prospective enrollment of participants and all data were publicly available.

### Data Source

Data were obtained from the Adverse Pregnancy Outcomes Reporting System (APORS) from the Illinois Department of Public Health. APORS collects data on neonates born with birth defects or other abnormal conditions. All Illinois hospitals with a licensed labor and delivery unit report to APORS, including rural hospitals and birthing hospitals in St Louis, Missouri, which serve patients residing in southern Illinois. APORS additionally abstracts birth and death certificate data, engages in continual hospital training and reviews each case submitted to ensure data quality, case completeness, and timeliness. These activities assure APORS is the most complete data source on birth defects in Illinois.^13^

### Study Design and Population

This is a cross-sectional analysis of all live-born infants with any CHD reported to the Illinois Department of Public Health, born between July 1, 2013, and December 31, 2021. We identified these infants using the *International Statistical Classification of Diseases, Tenth Revision, Clinical Modification *(*ICD-10-CM*) diagnosis codes (eTable 1 in Supplement 1), which were being used by APORS prior to their clinical implementation in 2015. We chose 2013 as the start year as 2013 represents the year that pulse oximetry screening for critical CHD was introduced by the Illinois Department of Public Health, as differences in the administration of pulse oximetry screening could have biased our analysis. We chose 2021 as the end year because it contained the most complete data at the time of data acquisition. Infants with acquired heart disease (eg, endocarditis), isolated patent foramen ovale or patent ductus arteriosus, unknown birth hospital or prenatal care status were excluded.

### Outcome and Exposure Variables

The primary outcome variable was delivery at a cardiac center, a binary measure of whether or not the neonate was delivered at a cardiac center. A cardiac center was defined as a center with capabilities to perform the highest complexity congenital heart operations per The Society of Thoracic Surgeons, which collects operative information from 95% of all CHD operations in the US and Canada.^14^ Centers with capabilities for cardiac catheterization, but not cardiac operations, were not defined as cardiac centers for the purposes of this analysis, even though these centers have advanced care capabilities for children with CHDs. The dataset included 125 birth hospitals, of which 5 cardiac centers met this definition. Of these 5 centers, 3 were in Illinois, and 2 were in St Louis, Missouri.

Two exposure variables were derived from the Kotelchuck Index—a validated measure of prenatal care adequacy that is calculated from birth certificate data.^15,16^ The Kotelchuck Index categorizes prenatal care adequacy into 5 levels based on prenatal care initiation and prenatal visit frequency—2 components of prenatal care adequacy with different effects on outcomes.^16^ These levels include no prenatal care, inadequate (initiated after the fourth month or less than 50% of recommended visits), intermediate (50% to 79% of recommended visits), adequate (80% to 109% of recommended visits), and adequate plus (110% or more of recommended visits). In this cohort, inadequate care primarily reflected delayed care because 88% of those with inadequate care had initiated prenatal care after the fourth month.

While Kotelchuck has previously described how prenatal care initiation and prenatal visit frequency measure different aspects of prenatal care adequacy,^16^ we identified an additional reason that prenatal care adequacy could not be analyzed as a single, 5-category exposure variable for this analysis. Specifically, on-time prenatal care (ie, intermediate, adequate, or adequate plus) could not be combined with inadequate prenatal care to measure prenatal care initiation because those with on-time care had significantly lower rates of severe CHD, noncardiac comorbidities, and rates of delivery at cardiac centers, which may be due to higher termination or fetal loss rates (eTable 2 in Supplement 1). This specific type of selection bias, survival bias due to fetal loss among neonates with severe birth defects, has been previously well-described.^17,18^

Therefore, 2 binary exposure variables were defined from the Kotechuck index. The first exposure variable was prenatal care initiation (ie, no prenatal care vs inadequate [delayed] prenatal care). The second exposure variable was the prenatal visit frequency (ie, intermediate prenatal care vs adequate or adequate plus care). Since these categories reflect prenatal care initiation before the fourth month, the second exposure variable specifically measured the effect of increasing visit frequency.

### Covariates

Additional covariates for the birth parent included gravidity (nulligravida, multigravida); education (less than high school, high school diploma, some college, bachelor’s degree or higher); race or ethnicity (Hispanic, non-Hispanic Asian, non-Hispanic Black, non-Hispanic White, multiracial or other [ie, not conforming to the aforementioned categories]); payor (public, other); and straight-line distance to the nearest cardiac center (analyzed distance in quartiles to account for nonlinear associations). APORS abstracts race and ethnicity data from birth certificates, which are self-reported by the birthing parent of the neonate. Race and ethnicity were necessary covariates because they were associated with both exposure (prenatal care initiation or visit frequency) and outcome (birth hospital level of care). Distance was calculated as the straight-line distance between the maternal address and the address of the nearest cardiac center. We defined other payors as those with private insurance, those with military or Tricare insurance, or those who were self-pay, because these individuals are likelier to have a stable income than individuals with Medicaid, Medicare, or charity care (public payor).

Additional covariates for the neonate included the number of comorbidities (0, 1, 2, 3, 4 or more); plurality (singleton, multifetal gestation); and birth weight (continuous). Neonatal comorbidities were coded using the previously validated Pediatric Complex Chronic Condition (CCC) System, Version 3.^19^ A neonate with 2 gastrointestinal CCCs was counted as having 1 comorbidity, but a neonate with a gastrointestinal and pulmonary CCC was counted as having 2 comorbidities. Cardiac CCCs were excluded. The comorbidity count was analyzed as a categorical rather than continuous variable due to nonlinear associations between comorbidity count and the outcome.

Several variables in the APORS dataset were tested but excluded from the final models. These included parity (excluded due to collinearity with gravidity); gestational age (excluded due to collinearity with birth weight); census-tract level neighborhood deprivation (excluded due to collinearity with distance); census-tract level urbanicity (defined by the US Census Bureau,^20^ excluded due to collinearity with distance); marital status (excluded due to collinearity with payor); and neonatal sex (excluded due to lack of association with exposures or outcome).

### Statistical Analysis and Sensitivity Analysis

Statistical analysis was performed from November 2024 to May 2025. Standard descriptive statistics were performed, along with bivariate comparisons using Kruskal-Wallis, χ^2^, or *t* tests. All statistical testing was 2-sided, with significance defined at α = .05. We used multivariable linear probability regression models with robust standard errors to estimate associations between each exposure and outcome. For all models, we included a fixed effect for birth year to account for era effects (eg, the COVID-19 pandemic).

#### Model Specifications

Our final model specifications were:

Equation 1: probability of delivery at cardiac center = β0 + β1 × prenatal care intiation + β2 × neonate comorbidities + β3 × birth weight + β4 × gravidity + β5 × birthing parent race and ethnicity + β6 × education + β7 × payor + β8 × distance from cardiac center + β9 × birth year fixed effect + ε_1_

Equation 2: probability of delivery at cardiac center = β0 + β1 × frequency of prenatal visits + β2 × neonate comorbidities + β3 × birth weight + β4 × plurality + β5 × gravidity + β6 × birthing parent race and ethnicity + β7 × education + β8 × distance from cardiac center + β9 × birth year fixed effect + ε_1_.

Each equation was then stratified by CHD severity (mild, moderate, or severe), with these 3 groups determined by previous expert consensus^21^: severe if neonatal intervention was required for survival (eg, hypoplastic left heart syndrome), moderate if intervention was likely required before the first year of life but after the neonatal period (eg, tetralogy of Fallot), and mild if surgical intervention is not required within 1 year of life, if at all (eg, septal defects, eTable 1 in Supplement 1). This stratification was necessary because mild CHDs do not require delivery at a cardiac center, even if high-level neonatal intensive care is required for noncardiac comorbidities like severe prematurity. In contrast, severe CHDs require intervention at a cardiac center, typically within the first week of life. This yielded 6 regressions for the main result. Plurality was excluded from Equation 1 and payor was excluded from Equation 2 to achieve parsimony (ie, neither affected the association between exposure and outcome, nor improved model fit [R^2^]).

#### Sensitivity Analyses

We performed several sensitivity analyses. First, to test whether the large sample size was responsible for type I error, we performed an analysis using bootstrapping with replacement. Second, to test the robustness of our CHD severity definitions, we removed those with Ebstein anomaly (n = 71) from the severe CHD group, because the severity of clinical presentation among those with Ebstein anomaly is highly variable. To test whether isolated coronary anomalies (n = 103) were appropriately classified as mild CHD, we repeated regressions after reclassifying them as moderate disease. All analyses were conducted using Stata version 18 (StataCorp).

## Results

### Cohort Characteristics

During the study period, 12 113 neonates with CHD were reported to APORS (2713 Hispanic neonates [22.4%]; 428 non-Hispanic Asian [3.5%]; 2409 non-Hispanic Black [19.9%]; 5886 non-Hispanic White [48.6%]). Of these, 272 neonates (2.3%) had no prenatal care, 1617 (13.4%) had inadequate prenatal care, 1304 (10.8%) had intermediate prenatal care, 4217 (34.8%) had adequate prenatal care, and 4703 (38.8%) had adequate plus prenatal care. Also, 1579 neonates (13.0%) had severe CHD, 1694 (14.0%) had moderate CHD, and 8840 (73.0%) had mild CHD (Table 1). In this cohort, 3076 neonates (25.4%) were born at a cardiac center.

**Table 1.** Cohort Characteristics Stratified by CHD Severity^a^

Characteristics	Neonates, No. (%)				
	Total (n = 12 113)	Mild CHD (n = 8840)	Moderate CHD (n = 1694)	Severe CHD (n = 1579)	*P* value
Kotelchuck index^b^					
None	272 (2.3)	201 (2.3)	38 (2.2)	33 (2.1)	<.001
Inadequate	1617 (13.4)	959 (10.9)	255 (15.1)	403 (25.5)	
Intermediate	1304 (10.8)	994 (11.2)	164 (9.7)	146 (9.3)	
Adequate	4217 (34.8)	3139 (35.5)	576 (34.0)	502 (31.8)	
Adequate plus	4703 (38.8)	3547 (40.1)	661 (39.0)	495 (31.4)	
Delivery at cardiac center^c^					
No	9037 (74.6)	7011 (79.3)	1195 (70.5)	831 (52.6)	<.001
Yes	3076 (25.4)	1829 (20.7)	499 (29.5)	748 (47.4)	
Birthing parent characteristics					
Gravidity					
Nulligravida	3349 (27.7)	2538 (28.7)	432 (25.5)	379 (24.0)	<.001
Multigravida	8012 (66.1)	5918 (67.0)	1134 (66.9)	960 (60.8)	
Missing	752 (6.2)	384 (4.3)	128 (7.6)	240 (15.2)	
Race and ethnicity					
Hispanic	2713 (22.4)	2049 (23.2)	320 (18.9)	344 (21.8)	<.001
Non-Hispanic Asian	428 (3.5)	304 (3.4)	58 (3.4)	66 (4.2)	
Non-Hispanic Black	2409 (19.9)	1758 (19.9)	327 (19.3)	324 (20.5)	
Non-Hispanic White	5886 (48.6)	4199 (47.5)	893 (52.7)	794 (50.3)	
Other or multiple race^d^	677 (5.6)	530 (6.0)	96 (5.7)	51 (3.2)	
Level of education					
Less than high school	1545 (12.9)	1135 (13.0)	206 (12.3)	204 (13.1)	.003
High school diploma	2884 (24.2)	2091 (24.0)	391 (23.3)	402 (25.8)	
Some college	3216 (26.9)	2300 (26.4)	450 (26.9)	466 (29.9)	
Bachelor’s degree or more	4296 (36.0)	3180 (36.5)	628 (37.5)	488 (31.3)	
Payor					
Other^e^	7138 (59.1)	5237 (59.4)	1031 (60.9)	870 (55.10)	.001
Public	4947 (40.9)	3577 (40.6)	661 (39.1)	709 (44.9)	
Distance to nearest cardiac center, median (IQR), miles	20.2 (7.3-39.1)	19.9 (7.06-38.24)	23.2 (8.4-44.8)	19.7 (7.5-39.0)	<.001
Neonate characteristics					
Plurality					
Single gestation	11091 (91.6)	8069 (91.3)	1538 (90.8)	1484 (94.0)	.001
Multifetal gestation	1022 (8.4)	771 (8.7)	156 (9.2)	95 (6.0)	
Birth weight, mean (SD), kg	2.8 (1.0)	2.8 (1.0)	2.8 (1.0)	3.0 (0.9)	<.001
No. of comorbidities^f^					
None	5713 (47.2)	4738 (53.6)	544 (32.1)	431 (27.3)	<.001
1	2504 (20.7)	1601 (18.1)	457 (27.0)	446 (28.3)	
2	1588 (13.1)	1016 (11.5)	279 (16.5)	293 (18.6)	
3	994 (8.2)	652 (7.4)	167 (9.9)	175 (11.1)	
≥4	1314 (10.9)	833 (9.4)	247 (14.6)	234 (14.8)	

^a^ Categorizations of CHD severity can be found in eTable 1 in Supplement 1.

^b^ Validated measure of prenatal care adequacy. The 5 categories are no prenatal care, inadequate (prenatal care initiated after the fourth month or less than 50% of expected prenatal visits received), intermediate (50% to 79% of recommended visits received), adequate (80% to 109% of recommended visits), and adequate plus (110% or more of recommended visits) prenatal care.

^c^ Cardiac center is 1 of 7 birth hospitals in Illinois and surrounding states that perform the highest-complexity neonatal cardiac operations per The Society of Thoracic Surgeons.

^d^ Other or multiple race denotes those who identify as multiracial, unknown race, or report a race or ethnicity combination that is not Hispanic, non-Hispanic Asian, non-Hispanic Black, or non-Hispanic White.

^e^ Other payor includes private insurance, military or Tricare, and self-pay. Public payor includes Medicaid, Medicare, or charity.

^f^ Comorbidities based on the Pediatric Complex Chronic Condition system, Version 3.

When comparing cohort characteristics across prenatal care categories, those with inadequate (delayed initiation after 4 months) prenatal care were likelier than those with intermediate and adequate or adequate plus (initiation before 4 months) care to be publicly insured (866 of 1617 [53.8%] vs 639 of 1304 [49.1%] and 3257 of 8920 [36.6%]; *P* < .001), and have a high school diploma or less (787 of 1617 [49.6%] vs 558 of 1304 [43.7%] and 2906 of 8920 [33.0%]; *P* < .001). Those with inadequate prenatal care also had a higher prevalence of severe CHD (403 of 1617 [24.9%] vs 146 of 1304 [11.2%] and 997 of 8920 [11.2%]; *P* < .001) and lower prevalence of comorbidity-free neonates compared with those with intermediate and adequate or adequate plus care (39.8% vs 48.4%-52.1%; *P* < .001.). Those with inadequate prenatal care were also likelier to deliver at a cardiac center compared with those with intermediate and adequate or adequate plus care (644 of 1617 [39.8%] vs 357 of 1304 [27.4%] and 2030 of 8920 [22.0%]; *P* < .001) (eTable 2 in Supplement 1).

### Adjusted Associations of Prenatal Care Initiation and Delivery at a Cardiac Center

After adjusting for birthing parent and neonate characteristics (Equation 1), inadequate prenatal care, compared with no prenatal care, was associated with a higher probability of delivery at a cardiac center across all CHD severity groups, with the increasing strength of association by CHD severity (mild CHD: 10.5 [95% CI, 4.7-16.2] percentage points; *P* < .001; moderate CHD: 17.9 [95% CI, 0.9-34.8] percentage points; *P* = .04; severe CHD: 30.2 [95% CI, 13.6-46.9] percentage points; *P* < .001) (Table 2).

**Table 2.** Adjusted Associations Between Prenatal Care Initiation and Probability of Delivery at a Cardiac Center

Exposure	Difference in probability of delivery at a cardiac center		
	Mild CHD (n = 1122)	Moderate CHD (n = 289)	Severe CHD (n = 427)
Prenatal care initiation^a^			
None	[Reference]	[Reference]	1 [Reference]
Inadequate	0.105 (0.047 to 0.162)	0.179 (0.009 to 0.348)	0.302 (0.136 to 0.469)^b^
Neonate comorbidities, No.^c^			
None	[Reference]	[Reference]	[Reference]
1	0.039 (−0.020 to 0.097)	−0.071 (−0.218 to 0.077)	0.039 (−0.069 to 0.148)
2	0.072 (−0.001 to 0.145)	0.013 (−0.147 to 0.173)	0.023 (−0.100 to 0.145)
3	0.151 (0.060 to 0.243)	−0.026 (−0.251 to 0.198)	−0.081 (−0.235 to 0.073)
≥4	0.145 (0.055 to 0.235)	0.042 (−0.125 to 0.209)	0.007 (−0.145 to 0.160)
Birth weight, kg	−0.006 (−0.034 to 0.021)	0.018 (−0.045 to 0.080)	0.059 (−0.001 to 0.120)
Gravidity			
Nulligravida	[Reference]	[Reference]	[Reference]
Multigravida	−0.027 (−0.079 to 0.026)	0.062 (−0.077 to 0.201)	0.031 (−0.083 to 0.144)
Missing	−0.048 (−0.156 to 0.060)	0.313 (0.141 to 0.486)	0.158 (0.028 to 0.288)
Birthing parent race or ethnicity			
Hispanic	[Reference]	[Reference]	[Reference]
Non-Hispanic Asian	−0.019 (−0.076 to 0.039)	−0.102 (−0.253 to 0.048)	−0.047 (−0.182 to 0.088)
Non-Hispanic Black	0.122 (0.056 to 0.188)	−0.015 (−0.180 to 0.150)	0.025 (−0.091 to 0.140)
Non-Hispanic White	−0.067 (−0.185 to 0.052)	0.142 (−0.165 to 0.448)	−0.156 (−0.357 to 0.045)
Other or multiple race^d^	0.027 (−0.094 to 0.148)	−0.133 (−0.445 to 0.179)	−0.268 (−0.557 to 0.021)
Level of education			
Less than high school	[Reference]	[Reference]	[Reference]
High school diploma	0.036 (−0.017 to 0.088)	−0.012 (−0.167 to 0.144)	0.047 (−0.094 to 0.189)
Some college	0.128 (0.065 to 0.192)	0.181 (0.009 to 0.353)	0.170 (0.038 to 0.303)
Bachelor’s degree or higher	0.223 (0.140 to 0.305)	0.235 (0.025 to 0.446)	0.230 (0.079 to 0.381)
Payor			
Other^e^	[Reference]	[Reference]	[Reference]
Public	−0.048 (−0.099 to 0.002)	−0.099 (−0.231 to 0.032)	−0.080 (−0.185 to 0.026)
Distance from nearest cardiac center in miles			
Quartile 1	[Reference]	[Reference]	[Reference]
Quartile 2	−0.154 (−0.219 to −0.090)	−0.184 (−0.326 to −0.042)	−0.027 (−0.142 to 0.088)
Quartile 3	−0.271 (−0.337 to −0.205)	−0.225 (−0.371 to −0.079)	−0.078 (−0.208 to 0.052)
Quartile 4	−0.307 (−0.380 to −0.235)	−0.270 (−0.427 to −0.113)	0.091 (−0.030 to 0.213)

^a^ Based on the Kotelchuck index, a validated measure of prenatal care adequacy. Inadequate prenatal care primarily reflects initiation of prenatal care after the fourth month. Intermediate, adequate, and adequate plus prenatal care are not included in the definition of prenatal care initiation due to selection bias in these prenatal care categories (eTable 2 in Supplement 1).

^b^ Statistically significant values. Coefficient interpretation: among neonates with severe CHD, those with inadequate prenatal care had, on average, a 30.2 (95% CI, 13.6 to 46.9) percentage point higher adjusted probability of delivery at a cardiac center than those without prenatal care.

^c^ Comorbidities based on the Pediatric Complex Chronic Condition (CCC) system, Version 3.

^d^ Other or multiple race denotes those who identify as multiracial, unknown race, or report a race or ethnicity combination that is not Hispanic, non-Hispanic Asian, non-Hispanic Black, or non-Hispanic White.

^e^ Private payor includes private insurance, military/Tricare, and self-pay. Public payor includes Medicaid, Medicare, or charity.

### Adjusted Associations of Prenatal Visit Frequency and Delivery at a Cardiac Center

After adjusting for birthing parent and neonate characteristics (Equation 2), among neonates with moderate and severe CHD, there was no significant association between prenatal visit frequency and probability of delivery at a cardiac center (Table 3). Among neonates with mild CHD, adequate plus (110% or more of visits) prenatal care was associated with a 6.7 (95% CI, 4.0-9.4) percentage points decrease in the probability of delivery at a cardiac center compared with intermediate (50% to 79% of visits) prenatal care (*P* < .001).

**Table 3.** Adjusted Associations Between Prenatal Visit Frequency and Probability of Delivery at a Cardiac Center

Exposure	Difference in probability of delivery at a cardiac center		
	Mild CHD (n = 7575)	Moderate CHD (n = 1385)	Severe CHD (n = 1133)
Prenatal visit frequency^a^			
Intermediate	[Reference]	[Reference]	[Reference]
Adequate	−0.020 (−0.047 to 0.007)	−0.033 (−0.108 to 0.043)	−0.042 (−0.133 to 0.048)
Adequate plus	−0.067 (−0.094 to −0.040)^b^	−0.046 (−0.121 to 0.030)	−0.076 (−0.167 to 0.014)
Neonate comorbidities, No.^c^			
None	[Reference]	[Reference]	[Reference]
1	0.037 (0.015 to 0.059)	−0.022 (−0.080 to 0.036)	0.035 (−0.038 to 0.109)
2	0.082 (0.054 to 0.109)	0.028 (−0.042 to 0.098)	−0.005 (−0.089 to 0.078)
3	0.110 (0.074 to 0.146)	−0.014 (−0.095 to 0.068)	0.021 (−0.078 to 0.120)
≥4	0.153 (0.118 to 0.187)	0.03 (−0.052 to 0.112)	0.089 (−0.003 to 0.181)
Birth weight, kg	−0.002 (−0.012 to 0.007)	−0.004 (−0.032 to 0.024)	−0.044 (−0.076 to −0.012)
Plurality			
Singleton	[Reference]	[Reference]	[Reference]
Multifetal gestation	0.028 (−0.002 to 0.059)	0.002 (−0.078 to 0.081)	−0.061 (−0.176 to 0.054)
Gravidity			
Nulligravida	[Reference]	[Reference]	[Reference]
Multigravida	−0.003 (−0.021 to 0.015)	−0.007 (−0.057 to 0.042)	0.004 (−0.061 to 0.070)
Missing	−0.082 (−0.123 to −0.040)	0.125 (0.018 to 0.233)	0.165 (0.060 to 0.269)
Birthing parent race or ethnicity			
Hispanic	[Reference]	[Reference]	[Reference]
Non-Hispanic Asian	0.014 (−0.028 to 0.057)	0.033 (−0.104 to 0.169)	0.089 (−0.070 to 0.247)
Non-Hispanic Black	−0.021 (−0.047 to 0.005)	−0.004 (−0.080 to 0.072)	−0.055 (−0.142 to 0.032)
Non-Hispanic White	0.106 (0.084 to 0.128)	0.128 (0.063 to 0.193)	0.123 (0.042 to 0.204)
Other or multiple race^d^	0.024 (−0.013 to 0.060)	−0.040 (−0.139 to 0.059)	−0.054 (−0.215 to 0.107)
Level of education			
Less than high school	[Reference]	[Reference]	[Reference]
High school diploma	0.044 (0.018 to 0.069)	0.089 (0.010 to 0.169)	−0.001 (−0.106 to 0.103)
Some college	0.059 (0.034 to 0.085)	0.032 (−0.043 to 0.107)	0.017 (−0.084 to 0.119)
Bachelor’s degree or higher	0.163 (0.136 to 0.190)	0.174 (0.094 to 0.254)	0.096 (−0.011 to 0.204)
Distance to nearest cardiac center, miles			
Quartile 1	[Reference]	[Reference]	[Reference]
Quartile 2	−0.265 (−0.293 to −0.238)	−0.293 (−0.360 to −0.226)	−0.098 (−0.180 to −0.017)
Quartile 3	−0.418 (−0.442 to −0.393)	−0.341 (−0.407 to −0.275)	−0.275 (−0.351 to −0.199)
Quartile 4	−0.433 (−0.458 to −0.409)	−0.335 (−0.404 to −0.266)	−0.207 (−0.292 to −0.122)

^a^ Based on the Kotelchuck index, a validated measure of prenatal care adequacy: intermediate (50% to 79% of recommended visits received), adequate (80% to 109% of recommended visits), and adequate plus (110% or more of recommended visits) prenatal care.

^b^ Statistically significant values. Coefficient interpretation: among neonates with mild CHD those with adequate plus prenatal care had, on average, a 6.7 (95% CI, 4-9.4) percentage point lower adjusted probability of delivery at a cardiac cent er than those with intermediate prenatal care.

^c^ Comorbidities based on the Pediatric Complex Chronic Condition system, Version 3.

^d^ Other or multiple race denotes those who identify as multiracial, unknown race, or report a race or ethnicity combination that is not Hispanic, non-Hispanic Asian, non-Hispanic Black, or non-Hispanic White.

### Sensitivity Analyses

The magnitude of associations changed minimally across all sensitivity analyses. The results can be found in eTables 3 and 4 in Supplement 1.

## Discussion

In this analysis of neonates born with CHDs in Illinois between 2013 and 2021, prenatal care initiation and prenatal visit frequency were associated with the probability of delivery at a cardiac center in distinct ways. Specifically, individuals with delayed (inadequate) prenatal care initiation were likelier to deliver at a cardiac center compared with those without prenatal care at all, with this probability increasing alongside CHD severity. Furthermore, among those with moderate or severe CHD who initiated prenatal care before the fourth month, more prenatal visits were not significantly associated with a difference in the probability of delivery at a pediatric cardiac center. However, among those with mild CHD, a higher prenatal visit frequency was associated with a lower likelihood of delivery at a cardiac center.

Although the association between prenatal CHD diagnosis and delivery hospital has been well-described,^5,11,22,23^ fewer studies have explored how prenatal care adequacy is associated with delivery location. Our analysis suggests that prenatal care may influence delivery location beyond the impact of prenatal diagnosis alone. Specifically, additional prenatal visits allow for further assessment of fetal cardiac anatomy and physiology over time, aiding in the selection of the most appropriate delivery location. For example, in tetralogy of Fallot with pulmonary stenosis, delivery at a cardiac center may be unnecessary if there is sufficient pulmonary blood flow (ie, pink tetralogy).^21^ Determination of sufficient pulmonary blood flow typically requires at least 1 follow-up fetal echocardiogram after initial prenatal diagnosis but before delivery.

Regionalization of CHD care is a debated topic, with most research focusing on the association between higher surgical volumes and improved outcomes, particularly among children requiring the most complex neonatal operations.^24,25,26^ Two models of regionalization are frequently proposed: consolidating the number of cardiac centers to increase surgical volume,^27,28,29^ or maintaining the current number of centers while referring complex cases to higher-volume centers.^30,31^ The success of both models depends on 2 conditions: (1) ensuring that neonates with severe CHDs are delivered at high-volume centers to optimize outcomes and (2) preserving resource capacity for those with severe CHD. By delivering neonates with nonsevere CHD at noncardiac centers, resource capacity can be preserved for the severe cases while also potentially limiting costly, low-value care^32,33^ among those who do not require neonatal cardiac intervention—or in the case of some mild CHDs, any intervention over their lifetime. Our findings highlight how both prenatal care initiation and visit frequency may contribute to improved regionalization of CHD care across the full spectrum of disease severity.

This analysis underscores that regionalization is a multifactorial process that requires considerations beyond the effects of surgical volume alone. Effective regionalization networks depend on referral patterns, interhospital coordination, financial incentives, and patient preferences.^8,34^ We have previously demonstrated that high-complexity CHD operations are less profitable than low-complexity operations under certain payment models,^35^ and that industry payments disproportionately favor high-volume centers.^36^ These findings highlight the potential financial implications of centralizing CHD care. In this analysis, we demonstrate that prenatal care use influences delivery location, which in turn affects referral patterns and the number of neonatal transfers for severe CHD. Advocates for regionalized CHD care must consider factors beyond center volume, including policies that support statewide perinatal regionalization networks^37^ and improve access to obstetric care.

### Limitations

This study has limitations, including the lack of obstetric clinical variables in our dataset, such as hypertensive disorders of pregnancy or body mass index, which may confound our findings. That said, the social determinants of health we included in this analysis, such as racial and ethnic identity, insurance status, and educational attainment, are associated with obstetric comorbidities^38,39^ and prenatal care adequacy.^40,41^ Another limitation was missing data for the gravidity and prenatal care variables. Results were similar when we substituted parity for gravidity. When comparing those with prenatal care data to those with missing data, the distributions of CHD severity were nearly identical. However, those with missing data were more likely to deliver at a cardiac center and were more likely to live closer to a cardiac center. This suggests that for those with missing data, delivery at a cardiac center may have been due to geographic proximity rather than CHD severity or prenatal care status itself. Third, moderate CHD represents a group that may or may not require delivery at a cardiac center, but* ICD-10-CM *codes are insufficiently granular to make this delineation. Therefore, this analysis cannot draw conclusions for the moderate CHD group. Finally, those infants whose mild CHD was diagnosed after discharge from their birth hospital would not have been captured by this dataset. These infants are more likely to have been delivered at centers where fewer cardiac diagnoses are made (ie, noncardiac centers). Therefore, excluding these patients may have led to overestimation of the association between prenatal care initiation and delivery at the cardiac center, and underestimation of the association between prenatal visit frequency and delivery at a noncardiac center for the mild CHD group. However, such findings would not change the implications of our current analysis.

## Conclusions

In this cross-sectional study of neonates with congenital heart defects born in Illinois between 2013 and 2021, initiating prenatal care, even when delayed, was associated with a greater chance of delivery at a cardiac center compared with no prenatal care for all CHD types. Higher prenatal visit frequency was associated with a higher chance of mild CHD delivering at noncardiac centers, which may improve the efficiency of resource allocation. While the CHD community may have a shared goal of regionalizing severe CHD to high-volume centers, operationalizing this goal requires an understanding of many factors, such as prenatal events.

## Notes

Supplement 1. **eTable 1. ***ICD-10* codes and CHD severity **eTable 2. **Covariate balance test: differences in cohort characteristics among those who initiated prenatal care before the fourth month **eTable 3. **Sensitivity analysis: checking robustness of standard errors using bootstrapping with replacement **eTable 4. **Sensitivity analysis: testing definitions of mild and severe CHD groups

Supplement 2.
Data Sharing Statement
