# Supplementary material for: Prenatal Care and Perinatal Regionalization for Congenital Heart Defects
Source: JAMA Netw Open. 2025 Nov 9;8(11):e2542135. doi: 10.1001/jamanetworkopen.2025.42135 (PMC12598512; doi:10.1001/jamanetworkopen.2025.42135)
Supplement: Supplement 1. — eTable 1. ICD-10 codes and CHD severity eTable 2. Covariate balance test: differences in cohort characteristics among those who initiated prenatal care before the fourth month eTable 3. Sensitivity analysis: checking robustness of standard errors using bootstrapping with replacement eTable 4. Sensitivity analysis: testing definitions of mild and severe CHD groups [file jamanetwopen-e2542135-s001.pdf]

Supplemental Online Content

Laternser C, Grobman WA, Albaro C, et al. Prenatal care and perinatal regionalization for congenital heart defects. *JAMA Netw Open*. 2025;8(10):e2542135.
doi:10.1001/jamanetworkopen.2025.42135

eTable 1. *ICD-10* codes and CHD severity
eTable 2. Covariate balance test: differences in cohort characteristics among those who initiated prenatal care before the fourth month
eTable 3. Sensitivity analysis: checking robustness of standard errors using bootstrapping with replacement
eTable 4. Sensitivity analysis: testing definitions of mild and severe CHD groups

This supplemental material has been provided by the authors to give readers additional information about their work.

© 2025 Laternser C et al. *JAMA Network Open*.

eTable 1. *ICD-10* codes and CHD severity

CHD severity	ICD-10 code	ICD-10 description
Severe	Q20.0	Common arterial trunk
Severe	Q20.1	Double outlet right ventricle
Severe	Q20.2	Double outlet left ventricle
Severe	Q20.3	Discordant ventriculoarterial connection
Severe	Q20.4	Double inlet ventricle
Severe	Q22.0	Pulmonary valve atresia
Severe	Q22.5	Ebstein anomaly
Severe	Q22.6	Hypoplastic right heart syndrome
Severe	Q23.4	Hypoplastic left heart syndrome
Severe	Q25.2	Atresia of aorta
Severe	Q25.21	Interruption of aortic arch
Severe	Q25.29	Other atresia of aorta
Severe	Q25.41	Absence and aplasia of aorta
Severe	Q25.5	Atresia of pulmonary artery
Severe	Q26.2	Total anomalous pulmonary venous connection
Moderate	Q20.5	Discordant atrioventricular connection
Moderate	Q21.3	Tetralogy of Fallot
Moderate	Q22.1	Congenital pulmonary stenosis
Moderate	Q22.4	Congenital tricuspid stenosis
Moderate	Q23.0	Aortic stenosis
Moderate	Q23.2	Mitral stenosis
Moderate	Q24.4	Subaortic stenosis
Moderate	Q24.3	Subpulmonic stenosis or pulmonary infundibular stenosis
Moderate	Q25.1	Coarctation of the aorta
Moderate	Q25.3	Supravalvar aortic stenosis
Moderate	Q25.42	Hypoplasia of aorta
Moderate	Q25.6	Pulmonary artery stenosis
Moderate	Q25.71	Coarctation of pulmonary artery
Mild	Q20.6	Isomerism of atrial appendages
Mild	Q20.8	Other congenital malformations of cardiac chambers and connections
Mild	Q20.9	Congenital malformation of cardiac chambers and connections, unspecified
Mild	Q21.0	Ventricular septal defect
Mild	Q21.1	Atrial septal defect
Mild	Q21.2	Atrioventricular septal defect
Mild	Q21.4	Aortopulmonary septal defect
Mild	Q21.8	Other congenital malformations of cardiac septa
Mild	Q21.9	Congenital malformation of cardiac septum, unspecified

© 2025 Laternser C et al. *JAMA Network Open*.

Mild	Q22.2	Congenital pulmonary valve insufficiency
Mild	Q22.3	Other congenital malformations of pulmonary valve
Mild	Q22.8	Other congenital malformations of tricuspid valve
Mild	Q22.9	Congenital malformation of tricuspid valve, unspecified
Mild	Q23.1	Congenital insufficiency of aortic valve
Mild	Q23.3	Congenital mitral insufficiency
Mild	Q23.8	Other congenital malformations of aortic and mitral valves
Mild	Q23.9	Congenital malformation of aortic and mitral valves, unspecified
Mild	Q24.0	Dextrocardia
Mild	Q24.2	Cor triatriatum
Mild	Q24.5	Malformation of coronary vessels
Mild	Q24.8	Other specified congenital malformations of heart
Mild	Q24.9	Congenital malformation of heart, unspecified
Mild	Q25.3	Supravalvular aortic stenosis
Mild	Q25.4	Other congenital malformations of aorta
Mild	Q25.40	Congenital malformation of aorta unspecified
Mild	Q25.43	Congenital aneurysm of aorta
Mild	Q25.44	Congenital dilation of aorta
Mild	Q25.45	Double aortic arch
Mild	Q25.46	Tortuous aortic arch
Mild	Q25.48	Anomalous origin of subclavian artery
Mild	Q25.49	Other congenital malformations of aorta
Mild	Q25.79	Other congenital malformations of pulmonary artery
Mild	Q25.8	Other congenital malformations of other great arteries
Mild	Q25.9	Congenital malformation of great arteries, unspecified
Mild	Q26.0	Congenital stenosis of vena cava
Mild	Q26.3	Partial anomalous pulmonary venous connection
Mild	Q26.4	Anomalous pulmonary venous connection, unspecified
Mild	Q26.8	Other congenital malformations of great veins
Mild	Q26.9	Congenital malformation of great vein, unspecified

© 2025 Laternser C et al. *JAMA Network Open*.

eTable 2. Covariate balance test: differences in cohort characteristics among those who initiated prenatal care before the fourth month *

	Inadequate N=1617	Intermediate N=1304	Adequate or Adequate plus N=8920	p-value
CHD severity (%) †				<0.001
mild	959 (59.3)	994 (76.2)	6686 (75.0)	
moderate	255 (15.8)	164 (12.6)	1237 (13.9)	
severe	403 (24.9)	146 (11.2)	997 (11.2)	
Delivery at CHD surgical center (%) ‡				<0.001
no	973 (60.2)	947 (72.6)	6890 (77.2)	
yes	644 (39.8)	357 (27.4)	2030 (22.0)	
**Birthing Parent Characteristics**				
Gravidity (%)				<0.001
nulligravida	423 (26.2)	324 (24.9)	2553 (28.6)	
multigravida	1001 (61.9)	913 (70.0)	5901 (66.2)	
missing	193 (11.9)	67 (5.1)	466 (5.2)	
Race and Ethnicity (%)				<0.001
Hispanic	435 (26.9)	301 (23.1)	1919 (21.5)	
non-Hispanic White	607 (37.5)	547 (42.0)	4654 (52.2)	
non-Hispanic Asian	63 (3.9)	38 (2.9)	324 (3.63)	
non-Hispanic Black	437 (27.0)	356 (27.3)	1494 (16.8)	
other or multiple race	75 (4.6)	62 (4.8)	529 (5.9)	
Level of education (%)				<0.001
less than high school	297 (18.7)	204 (16.0)	968 (11.0)	
high school diploma	490 (30.9)	354 (27.7)	1938 (22.0)	
some college	396 (25.0)	350 (27.4)	2401 (27.2)	
bachelor’s degree or higher	402 (25.4)	370 (29.0)	3508 (39.8)	
Payor (%) §				<0.001
private	745 (46.2)	662 (50.9)	5649 (63.4)	
public	866 (53.8)	639 (49.1)	3257 (36.6)	
Mean age, in years (SD)	2.9 (0.8)	3.0 (0.9)	2.8 (1.0)	<0.001
Marital status				<0.001
unmarried	874 (54.1)	657 (50.4)	3414 (38.3)	
married	743 (45.9)	647 (49.6)	5506 (61.7)	
Median distance to nearest cardiac center, in miles [IQR]	17.0 [7.1,36.8]	15.3 [6.1,34.6]	21.5 [7.8,34.6]	<0.001
Residential urbanicity ¶				0.049
urban	1435 (88.7)	1165 (89.3)	7757 (87.0)	
urban cluster	113 (7.0)	86 (6.6)	756 (8.5)	
rural	69 (4.3)	53 (4.1)	406 (4.6)	
**Neonate Characteristics**				
Plurality (%)				<0.001
singleton	1527 (94.4)	1251 (96.0)	8059 (90.3)	

© 2025 Laternser C et al. *JAMA Network Open*.

multifetal gestation	90 (5.6)	53 (4.1)	861 (9.7)	
Mean birth weight, in kg (SD)	2.9 (0.8)	3.0 (0.9)	2.8 (1.0)	<0.001
Prematurity				<0.001
<37 weeks	413 (25.5)	309 (23.7)	3215 (36.0)	
≥ 37 weeks	1204 (74.5)	995 (76.3)	5705 (64.0)	
Number of comorbidities **				<0.001
none	644 (39.8)	679 (52.1)	4320 (48.4)	
1	374 (23.1)	253 (19.4)	1808 (20.3)	
2	244 (15.1)	175 (13.4)	1123 (12.6)	
3	166 (10.3)	69 (5.3)	732 (8.2)	
4+	189 (11.7)	128 (9.8)	937 (10.5)	

* As measured by the Kotelchuck index, a validated measure of prenatal care adequacy: intermediate (50-79% of recommended visits received), adequate (80-109% of recommended visits), and adequate plus (≥ 110% of recommended visits) prenatal care.
† Categorizations of CHD severity can be found in Supplemental Table 1.
‡ CHD surgical center is one of seven birth hospitals in Illinois and surrounding states that perform the highest-complexity neonatal cardiac surgeries per the Society of Thoracic Surgeons.
§ Private payor includes private insurance, military/Tricare, and self-pay. Public payor includes Medicaid, Medicare, or charity.
¶ Urbanicity definitions based on U.S. Census Bureau data, geocoded at the census tract level.
** Neonate comorbidities based on the Pediatric Complex Chronic Condition (CCC) system, Version 3.

© 2025 Laternser C et al. *JAMA Network Open*.

eTable 3. Sensitivity analysis: checking robustness of standard errors using bootstrapping with replacement

	Mild CHD	Moderate CHD	Severe CHD
Main result (Tables 2 and 3)			
Prenatal care initiation			
none	ref	ref	ref
inadequate	**0.105***	**0.179**	**0.302**
	**(0.047,0.162)**	**(0.009,0.348)**	**(0.136,0.469)**
Prenatal visit frequency			
intermediate	ref	ref	ref
adequate	-0.020	-0.033	-0.042
	(-0.047,0.007)	(-0.108,0.043)	(-0.133,0.048)
adequate plus	**-0.067**	-0.046	-0.076
	**(-0.094,-0.040)**	(-0.121,0.030)	(-0.167,0.014)
Bootstrapping with replacement			
Prenatal care initiation			
none	ref	ref	ref
inadequate	**0.105**	**0.179**	**0.302**
	**(0.046,0.163)**	**(0.013,0.344)**	**(0.138,0.467)**
Prenatal visit frequency			
intermediate	ref	ref	ref
adequate	-0.020	-0.033	-0.042
	(-0.046,0.006)	(-0.110,0.045)	(-0.131,0.046)
adequate plus	**-0.067**	-0.046	-0.076
	**(-0.092,-0.041)**	(-0.123,0.032)	(-0.167,0.014)

* Bolded values denote statistical significance. Coefficient interpretation: among neonates with mild CHD, those with inadequate prenatal care had, on average, a 10.5 higher percentage-point adjusted probability of delivery at a CHD surgical center than those without prenatal care.

© 2025 Laternser C et al. *JAMA Network Open*.

eTable 4. Sensitivity analysis: testing definitions of mild and severe CHD groups

	Mild CHD	Moderate CHD	Severe CHD
**Main results**			
Prenatal care initiation			
none	ref	ref	ref
inadequate	**0.105*** **(0.047,0.162)**	**0.179** **(0.009,0.348)**	**0.302** **(0.136,0.469)**
Prenatal visit frequency			
intermediate	ref	ref	ref
adequate	-0.020 (-0.047,0.007)	-0.033 (-0.108,0.043)	-0.042 (-0.133,0.048)
adequate plus	**-0.067** **(-0.094,-0.040)**	-0.046 (-0.121,0.030)	-0.076 (-0.167,0.014)
**Exclude Ebstein anomaly †**			
Prenatal care initiation			
none	N/A	N/A	**ref**
**inadequate***	N/A	N/A	**0.366** **(0.200,0.531)**
Prenatal care frequency			
intermediate	N/A	N/A	ref
adequate	N/A	N/A	-0.049 (-0.140,0.042)
adequate plus	N/A	N/A	-0.076 (-0.167,0.015)
**Reclassify isolated coronary anomalies ‡**			
Prenatal care initiation			
none	ref	ref	N/A
inadequate	**0.103** **(0.046,0.161)**	**0.171** **(0.001,0.334)**	N/A
Prenatal care frequency			
intermediate	ref	ref	N/A
adequate	-0.020 (-0.048,0.007)	-0.026 (-0.097,0.045)	N/A
adequate plus	**-0.065** **(-0.092,-0.038)**	-0.052 (-0.123,0.019)	N/A

* Coefficient interpretation: among those neonates with severe CHD, those with inadequate prenatal care had, on average, a 10.5 higher percentage-point adjusted probability of delivery at a CHD surgical center compared to those without prenatal care.
† Ebstein anomaly is classified as a severe CHD in the main analysis but excluded in this sensitivity due to its variable severity in postnatal presentation. Its exclusion does not affect the main results of the regressions for moderate and mild CHD.
‡ Reclassifies isolated coronary anomalies from mild CHD to moderate CHD. This reclassification does not affect the main results of the regressions for severe CHD.

© 2025 Laternser C et al. *JAMA Network Open*.
