# Supplementary material for: Prenatal Care and Perinatal Regionalization for Congenital Heart Defects
Source: JAMA Netw Open. 2025 Nov 9;8(11):e2542135. doi: 10.1001/jamanetworkopen.2025.42135 (PMC12598512; doi:10.1001/jamanetworkopen.2025.42135)
Supplement: Supplement 2. — Data Sharing Statement [file jamanetwopen-e2542135-s002.pdf]

Data Sharing Statement

Laternser. Prenatal Care and Perinatal Regionalization for Congenital Heart Defects. *JAMA Netw Open*. Published October 31, 2025. doi:10.1001/jamanetworkopen.2025.42135

Data
Data available: No
